# Purification and Characterization of Proteinaceous Thermostable α-Amylase Inhibitor from Sardinian Common Bean Nieddone Cultivar (*Phaseolus vulgaris* L.)

**DOI:** 10.3390/plants13152074

**Published:** 2024-07-26

**Authors:** Stefania Peddio, Sonia Lorrai, Tinuccia Dettori, Cristina Contini, Alessandra Olianas, Barbara Manconi, Antonio Rescigno, Paolo Zucca

**Affiliations:** 1 Department of Biomedical Sciences (DiSB), University Campus, Monserrato, 09042 Cagliari, Italy; stefania.peddio@unica.it (S.P.); sonia.lorrai@ulb.be (S.L.); dettorit@unica.it (T.D.); 2 Laboratoire de Production et de Biostimulation des Plantes cultivées, Faculté des Sciences, Université Libre de Bruxelles, 1050 Bruxelles, Belgium; 3 Department of Life and Environmental Sciences (DiSVA), University Campus, Monserrato, 09042 Cagliari, Italy; cristina.contini93@unica.it (C.C.); olianas@unica.it (A.O.); bmanconi@unica.it (B.M.)

**Keywords:** amylase, inhibitor, bean, enzyme, diabetes, phytohemagglutinin

## Abstract

The increasing need for new treatments for obesity and diabetes has led to the development of new drugs and food supplements that could reduce carbohydrate absorption. Many starch blockers, based on common bean proteinaceous inhibitors against α-amylase (α-AI), are already present on the market. The extraction and purification of α-amylase inhibitor from a promising common bean cultivar from Sardinia (Nieddone) is described, highlighting the unique value of the Nieddone cultivar, particularly for its inhibitory activity on digestive enzymes and its complete lack of a hemagglutination effect on human red blood cells. The purification of α-AI involved two chromatographic steps (IEC and SEC) and was essential for revealing certain properties of the inhibitor. The purified inhibitor has a tetrameric structure (α_2_β_2_) and a molecular weight of approximately 42 kDa, as determined by SEC and SDS-PAGE, confirming it as a lectin-like inhibitor. The identification of the α-AI sequence was obtained by bottom-up high-resolution mass spectrometry, which allowed us to identify a unique peptide from the α chain and six unique peptides from the β chains. α-AI exhibited an optimum temperature of around 40 °C and two pH optima at 5 and 6.5, respectively. Its remarkable stability at high temperatures was measured (approximately 25% of activity retained even after 5 h at 100 °C), whereas the raw extract lost its activity entirely after just 10 min at 90 °C. Thus, the purification process significantly enhances the thermal stability of α-AI. The demonstrated effectiveness of the purified α-AI against the α-amylase enzyme in pigs, humans and insects underscores the protein’s potential for treating obesity and diabetes, as well as for managing insect pests.

## 1. Introduction

The escalating global prevalence of metabolic disorders, particularly diabetes mellitus and obesity, has spurred intensive research into natural compounds capable of reducing carbohydrate absorption and exhibiting potential antidiabetic properties. Among these, α-amylase inhibitors (α-AIs) have garnered significant attention due to their role in modulating postprandial glucose levels [1].

These proteinaceous inhibitors interfere with the activity of α-amylase and α-glucosidase enzymes, reducing the breakdown of starch. This leads to a decreased digestibility of starch and a lower absorption of carbohydrates at the gut level [2].

Proteinaceous plant α-amylase inhibitors can be divided into several different classes: the Kunitz, the knottin-like, the thaumatin-like, the γ-thionin-like, the cereal and the lectin-like type [2,3]. Typically, in common beans, the latter type of inhibitors is present, known as α-AIs [4]. These proteins are produced by plants seeds, but they do not inhibit the activity of plant, fungal and bacterial α-amylases [5]. Their activity is effective against α-amylase in mammals, humans and insects [6].

α-AIs demonstrate dual potential. Firstly, they can be used as bioinsecticide or biopesticide to combat pest infestation. This has been demonstrated in the management of pests such as the cotton boll weevil (*Anthonomus grandis*) and *Callosobruchus maculatus*, with promising results [7,8]. Second, α-AIs can be utilized to create commercial starch blockers. In fact, numerous starch blockers, derived from α-AIs extracted from the common bean (*Phaseolus vulgaris*) are already on the market [2]. Many studies have in fact demonstrated their effectiveness against digestive enzymes extracted from different common bean cultivars [5,9,10,11].

However, despite its promising properties, the presence of antinutritional factors, such as hemagglutinin (PHA), in raw bean extract has raised questions about its safety and utility [12]. PHA is a protein that causes the agglutination of red blood cells in mammals. Many reports have documented haemagglutinin poisoning due to the consumption of raw common beans [13,14].

In this regard, different studies have investigated the inhibitory power of raw extracts from different common bean cultivars against the α-amylase and α-glucosidase enzyme as well as their hemagglutination power [4,15]. In a recent study conducted in our laboratories, ten Italian common bean cultivars were investigated for their α-amylase and α-glucosidase inhibiting activity and screened for the presence of PHA [4]. Among these cultivars, nine originated from Sardinia, the second-largest region in the Mediterranean Sea, known to be a biodiversity plant hotspot [16].

The Sardinian cultivar called Nieddone (ACC177) emerged as the most promising variety for its inhibitory activity against digestive enzymes and, above all, for the absence of hemagglutination power. Thus, the main aim of this study is to extract and characterize α-AI from the Nieddone cultivar and to evaluate its effectiveness against human, porcine and insect α-amylase.

This multifaceted approach aims to explore the therapeutic and agronomical potential of α-AIs but also to address safety concerns associated with antinutritional factors. Furthermore, the Nieddone cultivar is an almost forgotten common bean variety, with no scientific reports about its properties. Its valorization could help to preserve Sardinian biodiversity, safeguarding a sustainable agroecological model [17].

## 2. Results and Discussion

In a previous study, a screening of different common bean cultivars was conducted to identify the most promising variety for producing commercial food supplements [4].

The analyses revealed that the Nieddone cultivar is the most promising due to its significant inhibitory effect on digestive enzymes and the complete absence of hemagglutination activity.

**Figure 1:**
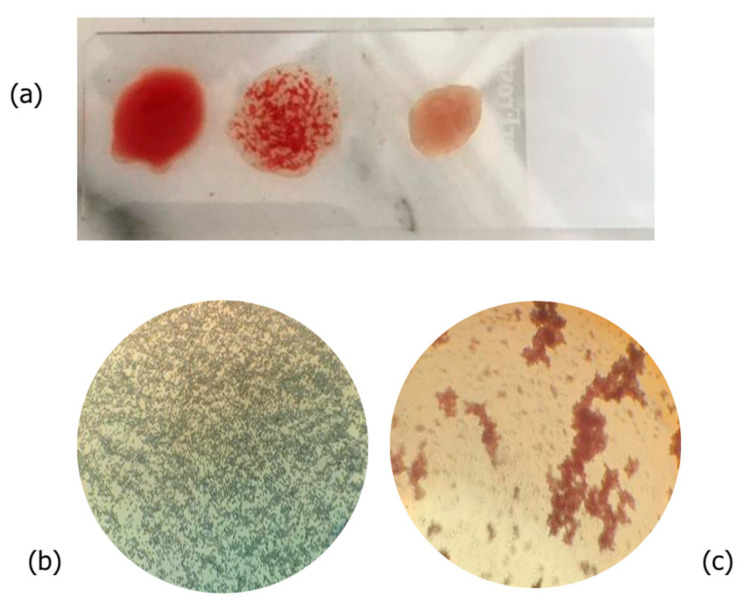
(**a**) An overview of different degrees of hemagglutination observed during the hemagglutination test of common bean extracts; (**b**) negative sample (Nieddone cv. raw extract); (**c**) positive sample (*P. vulgaris* Lamon cv raw extract).

This characteristic was confirmed by several investigations, which demonstrated that under the tested conditions, Nieddone raw extract exhibited no hemagglutination activity, even at the highest concentration tested, as shown in Figure 1. The same experiment was repeated after various purification steps (see below), confirming the absence of this antinutritional activity.

Given this evidence, the extraction, purification and characterization of α-AI isolated from the Nieddone cultivar were subsequently carried out.

### 2.1. Extraction and Low-Resolution Techniques for α-AI Purification

Initially, we employed low-resolution techniques for the inhibitor extraction to minimize production costs, resulting in an affordable food supplement [18], a key factor for a potential marketable nutraceutical product. 

The process began with grinding common bean seeds and suspending in 10 mM Bis-Tris buffer (pH 6.5, 0.1 M NaCl). We experimented with various extraction conditions, focusing on the impact of the protease inhibitor, extraction time and temperature on the α-AI activity, which are essential parameters of the process.

α-AI is produced as a pre-protein, and is proteolytically activated at Asn77. Therefore, we tested the addition of a well-known protease inhibitor (phenylmethylsulfonyl fluoride, PMSF) at 4 °C and 25 °C to check for any proteolytic activation during the early stages of purification.

We observed minimal proteolytic activation during the extraction, but any statistical difference was found in inhibitory activity, even after 24 h, regardless of whether PMSF was used. This suggests that no proteolytic activation occurs during the initial purification stages.

We recorded no significant increase in activity for extended incubation times (24 or 48 h, Figure S1). Similarly, no significant differences were observed between 4 °C and 25 °C. To prevent microbial contamination, the stirring was performed for 1 h in all experiments at 4 °C following established literature protocols [9,19].

The suspension obtained was then centrifuged, and the supernatant was collected.

Given that α-AI is a thermostable protein, we also heated the supernatant for 10 min at 60 and 70 °C, as previously suggested [20,21], to reduce total proteins while maintaining a high percentage of α-AI. This thermal treatment recovered almost all the inhibitory activity, but no increase in the specific activity was observed, leading us to discard this procedure.

Following the work of Bharadwaj, Gupta and Le Berre-Anton [9,19,20], we subjected the sample to various precipitation techniques, including 45–80% saturation with ammonium sulfate and cold organic solvents (ethanol and acetone) to enhance purification. We tested different ammonium percentages, but the best recovery extraction was obtained with the ethanol precipitation (Figure S2).

Consequently, we decided to use only ethanol precipitation as a low-cost purification technique. This method quickly increased sample purity, without requiring complex instrumentation or operator expertise. Additionally, it was cost-effective as the ethanol used could be efficiently distilled and reused for further purification cycles. The precipitate was then redissolved in the starting buffer and centrifugated before proceeding with further purification.

### 2.2. High-Resolution Techniques: Purification of α-AI

The α-AI from Nieddone was purified by two steps on a Q-Sepharose column (IEC) followed by chromatographic separation on a cross-linked agarose resin column (SEC).

In the first step, the redissolved ethanolic precipitate was loaded onto an anion exchange chromatography Q-Sepharose column which had been previously equilibrated with 10 mM Bis-Tris (pH 6.5, 0.1 M NaCl). It was then washed with buffer B (10 mM Bis-Tris pH 6.5, 1 M NaCl). The bound proteins were first eluted with a 20% step gradient in buffer B, and then with a 20–100% linear gradient with the same buffer B.

**Figure 2:**
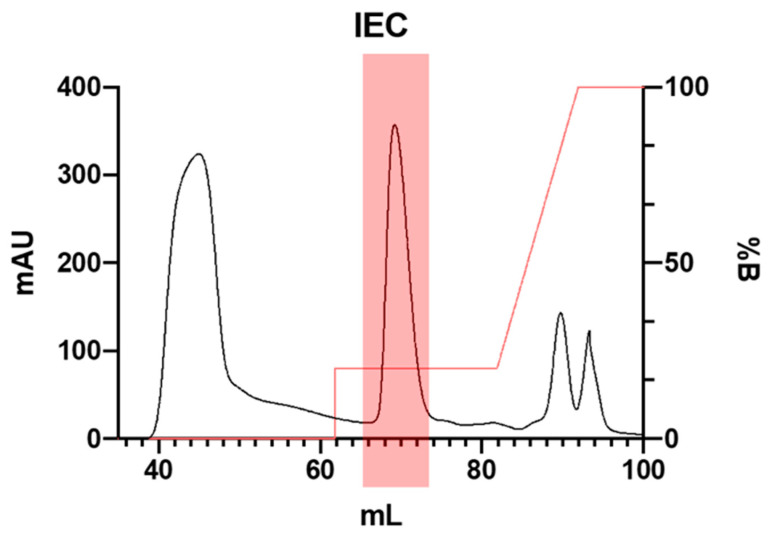
Elution profile of α-AI from *P. vulgaris* Nieddone cv on IEC Q-Sepharose column chromatography.

The elution pattern revealed a single peak with α-AI activity, which eluted at a low ionic strength (20% B), as depicted in Figure 2. To enhance the stability of the inhibitor, 5 mM CaCl2 was added to the active fractions. [22].

**Figure 3:**
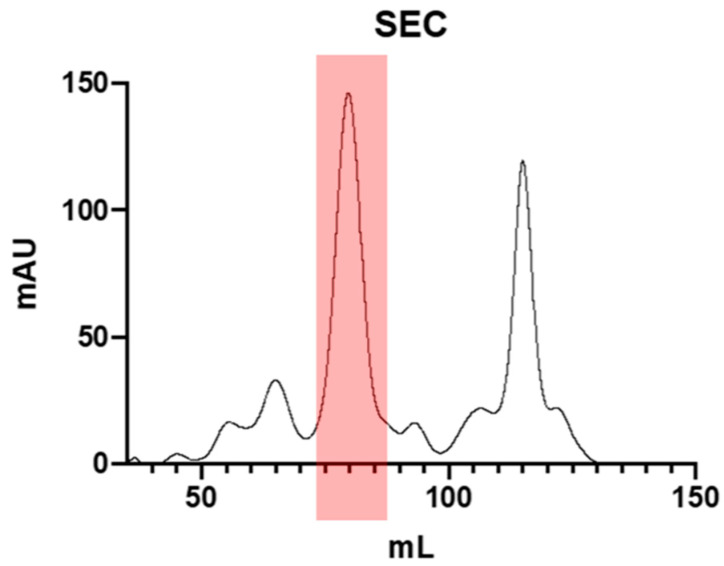
Purification profile of α-AI from *P. vulgaris* Nieddone cv on a SEC cross-linked agarose resin column.

Finally, the active fractions were collected and subjected to size exclusion chromatography (SEC) using a cross-linked agarose resin column. Binding proteins were eluted with an isocratic gradient using 10 mM bis-Tris (pH 6.5, 0.1 M NaCl, 5 mM CaCl_2_) buffer. A main peak showing biological activity was eluted around 80 mL Ve (Figure 3).

Traces of inhibitory activity were also detected in other eluted peaks, although this was quite unstable. This instability could potentially be attributed to some form of protein degradation or aggregations.

As indicated in Table 1, each purification step resulted in a significant increase in both specific activity and purification fold. Following the final purification step, the enzyme was purified more than 80-fold with a recovery rate of 33%. The specific activity of the purified α-AI (328 IAU/mg) was markedly higher than that of α-amylase from raw extract (4 IAU/mg). The lack of agglutination power was verified after each purification stage.

### 2.3. α-AI Identification

**Figure 4:**
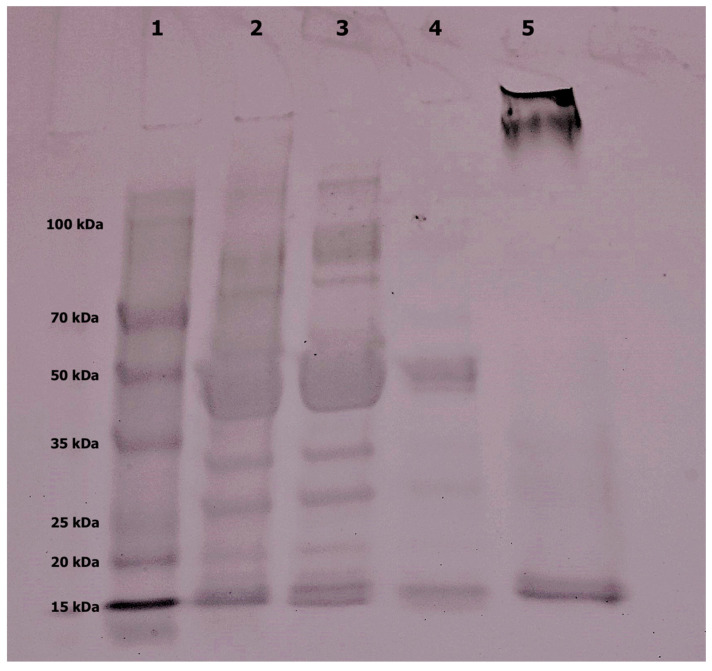
SDS-PAGE of α-AI from *P. vulgaris* Nieddone cv. during purification steps. Molecular weight standard is reported in line 1; α-AI raw extract is reported in Line 2; after the ethanolic precipitation in Line 3, after Q-Sepharose ion exchange chromatography in Line 4, after the size exclusion chromatography in Line 5.

All purification steps were analyzed by SDS-PAGE to assess the degree of purification. As depicted in Figure 4 (lane 5), the purified inhibitor displayed two bands close to the 15 kDa molecular standard.

Although a clear mobility difference between the two bands is not evident under the tested conditions, this evidence appears to be in line with the existence of two subunits, α and β chains, with theoretical molecular weights of 8 kDa and 15 kDa, respectively [23]). These data confirm that the purified protein is a lectin-like α-AI [3]. This type of protein is derived from a pre-protein that is proteolytically cleaved into two chains that associate to form the α_2_β_2_ tetramer [2].

To further confirm the identification of two chains of α-AI, the bands near the 15 kDa molecular standard in the SDS-PAGE (Figure 4, lane 5) were excised from the gel. Anyhow, they were too close to be separated, so they were collected as a single sample, cut into small pieces and subjected to destaining, reduction, alkylation and tryptic digestion before undergoing nano-RP-HPLC–high-resolution ESI-MS/MS analysis. 

**Figure 5:**
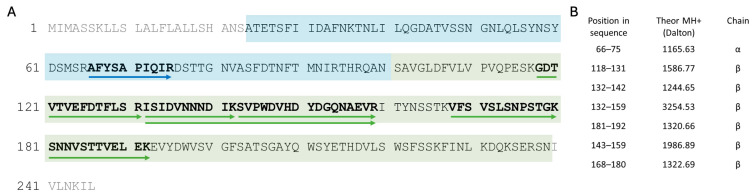
Identification of α-AI (UniprotKB code P02873) from *P. vulgaris* Nieddone cultivar. On the left (**A**), the entire sequence of α-AI is shown with α-chain highlighted by a blue box (position 24–100) and β-chain highlighted by a green box (position 101–239). In grey, the signal peptide (position 1–23) and the immature pro-peptide (position 240–246) of the pre-pro-sequence of α-AI are shown. Arrows indicate the tryptic peptides characterized by nano-RP-HPLC–high-resolution ESI-MS/MS analysis with the relative aminoacidic sequences in bold. The corresponding position in sequence, theoretical MH+ mass, and α or β chain are reported on the right (**B**).

As depicted in Figure 5, high-resolution mass spectrometry enabled us to confirm the presence of both the α-chain and β-chain of α-AI from the *P. vulgaris* Nieddone cultivar. The α-chain was characterized by a single unique peptide located at position 66–75 of the sequence, while six unique peptides from β-chain were observed. Figure 5 displays the sequence coverage (panel A), along with the exact position of each tryptic peptide attributed to α-AI, with the theoretical monoisotopic [M+1H]^+1^ mass of the peptides (panel B). The annotated MS/MS spectra from the Proteome Discoverer software for each tryptic peptide can be found in the Supplementary Material (Figure S3, panel A-G), where it is the *m*/*z* value of the ions with their charge selected for the MS/MS analysis is also reported. Overall, the 33.2% along the mature sequences of α-AI including both the α- and β-chain was identified based on the characterization of seven unique tryptic peptides (Figure 5).

Data from high-resolution mass spectrometry together with those from SDS-PAGE and SEC analyses allowed us to identify both the sequence (partially) and the molecular weight of α-AI from *Phaseolus vulgaris*, thus confirming the identity of α-AI and that it belongs to the lectin-like family. This assumption was also supported by the BLAST analysis performed on the seven unique tryptic peptides identified (Supplementary Figure S4).

In addition to the bands belonging to α and β subunits, SDS-PAGE revealed that some proteins were unable to penetrate the gel, resulting in an unresolved and undefined band near the well. This evidence could confirm the propensity of α-AI to form aggregates, as previously described in the literature [24], and as also suggested by SEC.

The tetrameric association of these two subunits, previously described [20], was confirmed by comparing the elution volume of the purified protein (Figure 3) with a mixture of molecular weight standards (Figure S5). A molecular weight of 42 kDa was estimated, consistent with the tetrameric α_2_β_2_ structure of the lectin-like α-AIs [20].

The literature records a significant variability in the molecular weight of *P. vulgaris* α-AI. A single subunit of 36 kDa was identified in *P. vulgaris* in 2008 [10], which aligns. with the monomeric structure described in *Mucuna pruriens* (25.6 kDa) [19] and *Secale cereale* (13.7 kDa) [25].

Different authors have described three subunits in *P. vulgaris* (α subunit ranged from 7.8 kDa to 15.5 kDa, β ranging from 14 kDa to 18.6 kDa and γ ranging from 22 kDa to 26.3 kDa) [9,26]. A study on the *P. vulgaris* inhibitor in 2011 described four subunits ranging from 14 to 20 kDa [27].

In contrast, two α-amylase inhibitors were identified in the tepary bean (*Phaseolus acutifolius* A. Gray) [28]. One of these (αAI-Pa2) was very similar to α-AI, being composed of α and β subunits, but it did not exhibit inhibitory activity against α-amylases of mammalian, bacterial or fungal origin, only against insect α-amylase.

Furthermore, two prominent amylase inhibitor activity bands (AI1 and AI2) were detected in the seeds of *Achyranthes aspera* [29]. The molecular weight of the purified inhibitor was approximately 6 kDa, and it is active against insect α-amylase.

However, the literature agrees on the tetrameric α_2_β_2_ structure for α-AI from *P. vulgaris*, with a total molecular weight of 44/45 kDa [2,30,31].

This variability could be attributed to the different techniques used for determining molecular weight or to the different cultivars and species analyzed.

#### 2.3.1. Prolonged Storage

**Figure 6:**
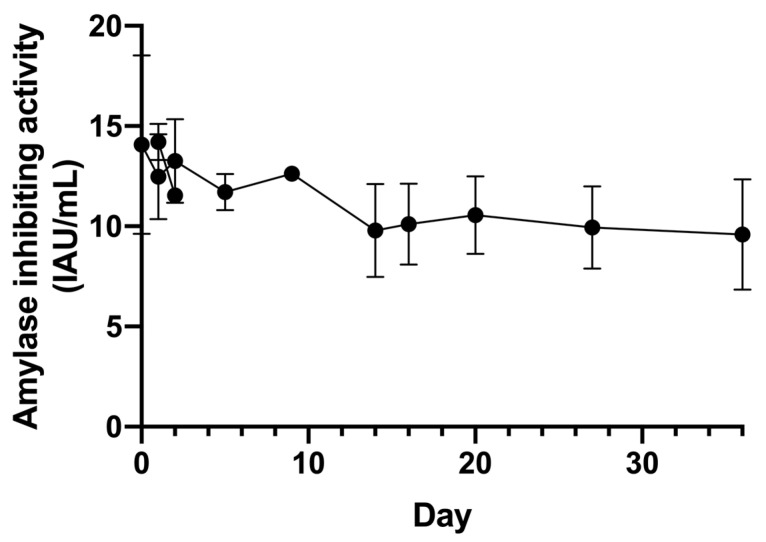
Trend over time of α-AI from Nieddone cv activity recorded with CNP-G3 assay. The time course was observed at regular intervals for 36 days (*p* < 0.05).

The purified α-AI was subsequently tested to assess its activity during extended storage. It was tested over a period of 36 days. The inhibitory activity appeared quite stable over time. After 36 days, only about 25/30% of its initial activity was lost (Figure 6). This stability is a desirable characteristic as it would allow the inhibitory power to be utilized during the production process of potential food supplements, and even the need for expensive and time-consuming solvent removal procedures (i.e., freeze-drying).

To the best of our knowledge, no similar storage stability has been studied thus far for α-AI. The long-lasting α-amylase and α-glucosidase inhibitory activity was studied, for instance, in white tea stored for 1, 3 and 5 years [32]. However, in this case, the inhibitory activity decreased with the reduction in total polyphenolic content, suggesting that these are likely not proteinaceous inhibitors.

#### 2.3.2. Activity of α-AI from Nieddone cv against Mammalian, Bacterial and Insect α-Amylases

The purified α-AI was subsequently evaluated for several operational characteristics, such as its inhibitory activity against the α-amylase enzyme from different organisms and the effect of pH and temperature conditions.

The activity of purified inhibitor was tested against four α-amylase enzymes from various sources, namely porcine pancreas, human saliva, *Bacillus subtilis* and *Tenebrio molitor*).

**Figure 7:**
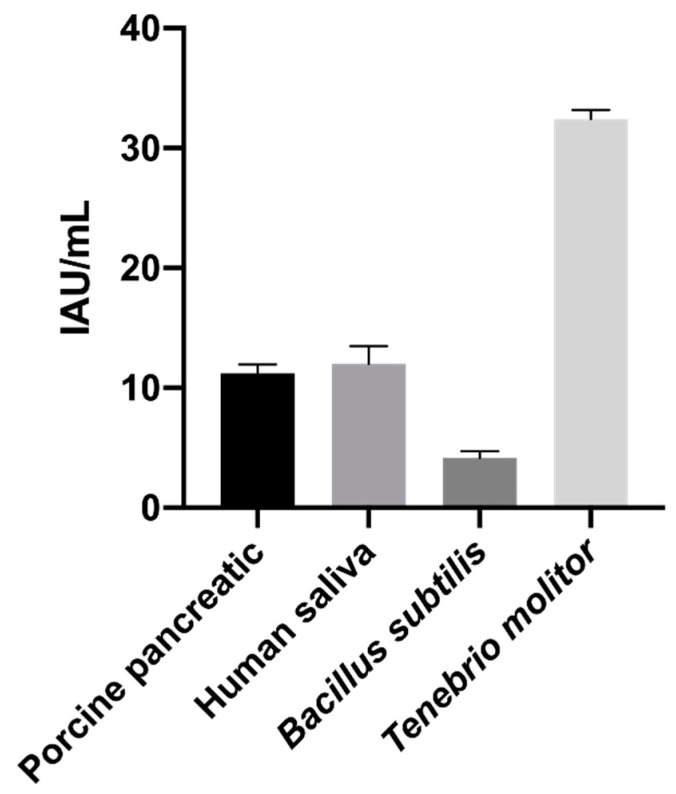
Inhibitory power of α-AI purified from Nieddone cv against pig, human, bacterial (*Bacillus subtilis*) and insect (*Tenebrio molitor*) α-amylase enzyme recorded with CNP-G3 assay (*p* < 0.05).

As depicted in Figure 7, the α-AI from the Nieddone cultivar exhibited the highest affinity against the insect α-amylase *Tenebrio molitor*. It was about three times more susceptible than human salivary amylase and porcine pancreatic α-amylase. The purified sample also demonstrated inhibitory activity against the α-glucosidase enzyme (3.8 ± 0.8 IGU/mL).

This result contrasts with observations made for the *Moringa oleifera* α-AI extract, which exhibited greater inhibitory activity against human α-amylase than *Callosobruchus maculatus* insect larvae [33]. Nevertheless the presence of inhibitory activity against both human and insect α-amylase confirms that the purified inhibitor corresponds to the α-AI1 isoform [34]. The high affinity of α-AI from *P. vulgaris* for insect α-amylase has also been observed in other species, including (*Tribolium castaneum*, *T. confusum* and *Sitophilus oryzae)* [35]. Recently, numerous authors have confirmed the role of the α-amylase inhibitors from various plant species against the α-amylase enzyme of insects [36,37]. This suggests that the administration of α-amylase inhibitors could be a valuable approach for biological pest control. Therefore, the α-AI from the Nieddone cultivar could play a crucial role in combating harmful pests that infect legumes of significant socioeconomic and nutritional importance.

#### 2.3.3. Time and Temperature Effects on α-AI

As previously mentioned, the inhibitory activity was recorded for 12 min with the CNP-G3 assay, which involved 10 min preincubation of the α-amylase enzyme/α-AI before addition of the substrate. Different preincubation times for common bean α-AI have been reported in the literature, with maximum inhibition achieved after 10 min [20], 40 min [21] and 120 min [38]. These differences could be due to the specific cultivar analyzed or the different assays used to record α-amylase activity. In contrast to the CNP-G3 assay used here, previous studies have employed the DNS assay.

We observed that the inhibitory activity of our purified inhibitor increased as preincubation time was extended (Figure S6), achieving complete inhibition with 120 min of preincubation, in accordance with Power and Whitaker [34].

The preincubation time is significant if the inhibitor is to be used for commercial purposes. These data suggest that a food supplement based on this inhibitor should be consumed two hours before a meal to achieve maximum inhibitory activity.

The effect of temperature on the α-AI optimum was studied by incubating the purified inhibitor at different temperatures ranging from 26 °C to 45 °C. It is well known that an increase in temperature exerts a dual effect of proteins, accelerating the reaction in accordance with the Arrhenius equation but also destabilizing the native state of proteins and thus inactivating them. The α-AI activity showed an optimum temperature of 40 °C.

Similar optimum values were also found for the α-amylase inhibitor extracted from *Mucuna pruriens* [19] and in kidney beans, where the optimum was reached at 37 °C [39,40].

This is a desirable condition for the use of an inhibitor in the production of dietary supplements, as it reaches its optimum at physiological body temperature.

Regarding the heat stability of α-AI, interesting data emerged from this study. It is known from the literature that plant proteinaceous inhibitors exhibit quite remarkable temperature stability. For example, the α-AI purified from *P. vulgaris* (Purola cultivar) was stable at 90 °C, preserving about 90% of its activity [5].

The α-amylase inhibitor purified from *Mucuna pruriens* (Fabaceae family) was found to be heat-stable, retaining about 80% of its activity after 30 min at 65 °C [19]. 

The thermal stability of α-amylase inhibitors was also observed in different varieties of *Triticum durum* [41]. In this case, both varieties were stable below 80 °C with the maximum inhibitory activity ranging from 40 to 50 degrees. When brought to the boil, the purified α-AI lost about 55–75% of its activity. Another purified proteinaceous α-amylase inhibitor extract from *Moringa oleifera* demonstrated heat stability, preserving about 50% of its activity for one hour at 70 °C [33].

**Figure 8:**
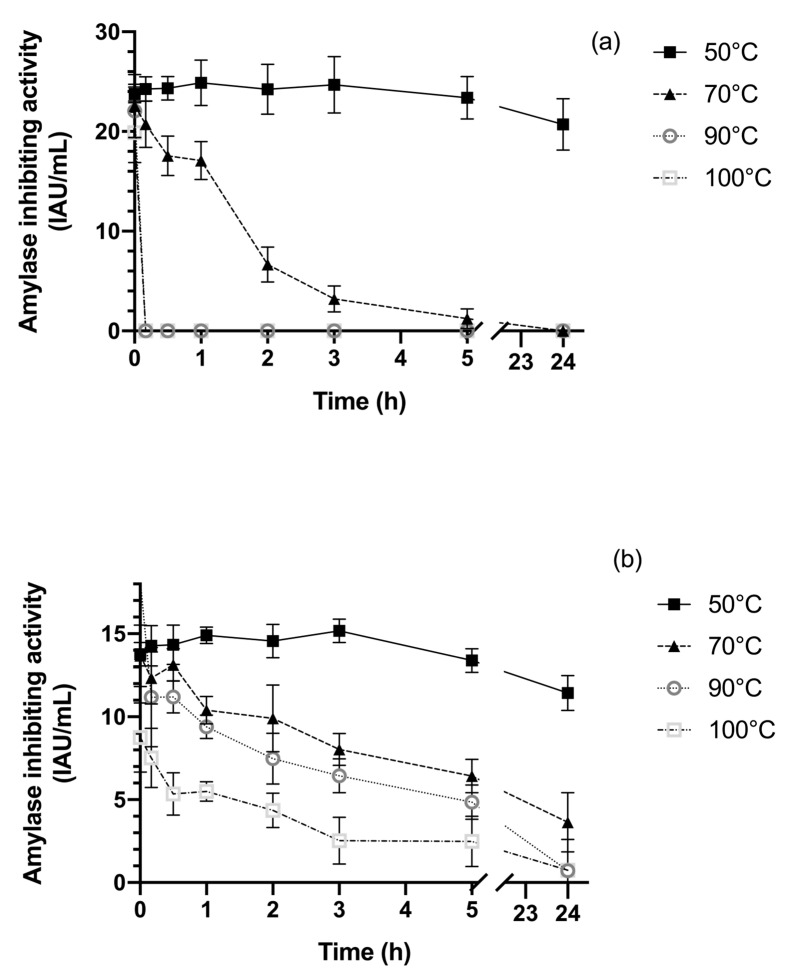
Effect of temperature on inhibitor stability. α-AI activity was assayed to different temperatures and the residual activity recorded at various time intervals. (**a**) The stability of the inhibitor from raw extract; (**b**) the stability of the purified extract from Nieddone cv (*p* < 0.05).

Our data seem to confirm this trend. In fact, we tested our purified sample at different temperatures ranging from 50 °C to 100 °C, and the inhibitory activity was recorded at different time intervals as shown in Figure 8b.

We also compared the temperature stability of the purified α-AI with that of the raw extract from the Nieddone bean (Figure 8a).

The two samples showed very different behaviors. Both were stable at 50 °C for up to 24 h (more than 90% activity retained). However, the inhibitory activity of the raw extract rapidly decreased at 70 °C after one hour, retaining about 75% of its activity. Its inhibitory activity was completely lost after 10 min at 90 °C, in accordance with previous observations [20,21,42].

On the other hand, the purified α-AI was remarkably more stable under the same conditions (Figure 8b), maintaining about 25% of its activity, even at 100 °C for 5 h. The inhibitory activity did not completely disappear even after 24 h, showing superior performance compared to other proteinaceous plant inhibitors [19,33].

It is possible for a purified protein to be more stable at a given temperature than a crude extract of the same protein. Crude extracts consist of a mixture of different proteins and other biomolecules, which can lead to instability due to interactions between these molecules. In contrast, a purified protein is isolated from these other molecules, which can enhance its stability. This desirable property has also been described in the Purola cultivar (*P. vulgaris*). In this case as well, the activity of the purified inhibitor was significantly more stable than the raw extract [5].

Our data show that the purified α-AI of the Nieddone cultivar is much more thermostable than the same protein present in the raw extract. This evidence suggests that the costs of purification could be justified by this increased stability. In fact, the resistance to high temperatures is a crucial parameter for producing inhibitor-enriched foods that need to be cooked before being consumed. This specificity would allow the production of palatable dietary food, especially when considering the production of pasta, particularly high-protein pasta. However, further investigations are needed to identify a procedure to further stabilize the inhibitory activity at higher temperatures.

#### 2.3.4. Effect of pH

In accordance with the literature, our data confirmed that the porcine pancreatic α-amylase enzyme exhibited a pH optimum at pH 5 (Figure S7). When the catalysis was performed in the presence of α-AI, using different 0.5 M McIlvaine buffers for incubation, two optima for inhibition were observed at pH 5 and 6.5.

Two optima (pH 5 and 6.9) were previously identified for the local Himalayan bean [9], while only one pH optimum (ranging from 4.5 to 6.9) was found in Japanese and kidney beans [20,21,22,43].

These differences could be attributed to the different experimental conditions or to the different α-amylase activity assay used (all the mentioned authors used the DNS assay).

In fact, a shift in the pH optimum was observed in the presence of different substrates [44].

Porcine pancreatic α-amylase hydrolyze starch has a pH optimum of 6.9, but the optimum shifts to 5.9 when a low molecular weight substrate like CNP-G3 is used, as in this study. However, we chose to perform the CNP-G3 assay at pH 6.5 in accordance with the literature [45]. This choice is related to the in vivo conditions of the α-amylase enzyme. In fact, in vivo, it works with starch as the substrate under conditions closer to neutrality than to an acidic environment.

#### 2.3.5. Kinetic Analysis

The kinetic analysis of the enzyme in the presence of the CNP-G3 substrate exhibited behavior typical of kinetics according to the Henri–Michaelis–Menten (H, M and M) equation. The K_M_ value was 0.85 mM and the V_max_ was 30.9 µM/min (Table 2). When the enzyme activity was conducted in the presence of inhibitor concentrations ranging between 0.25 and 0.50 µM, an increase in the of K_M_ value and a decrease in Vmax were observed. Specifically, at the concentration of 0.50 µM α-AI, a K_M_^app^ value of 1.10 and a V_max_^app^ = 21.8 were measured. 

**Figure 9:**
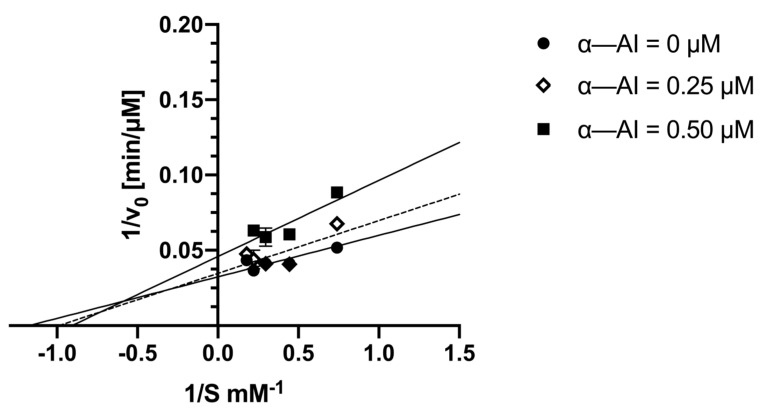
Lineweaver–Burk plots of α-amylase enzyme alone, inhibited with 0.25 µM α-AI and with 0.50 µM.

These values agreed with a non-competitive inhibition, as measured according to the plot of reciprocal doubles in the absence of the inhibitor and in the presence of partially inhibited catalysis (Figure 9). Specifically, the intercept of the lines in the second quadrant indicates a mixed-type inhibition where K_i_′ > K_i_. In our case, the values are 0.17 µM and 1.12 µM for K_i_ and K_i_′, respectively.

Previously, a mixed non-competitive inhibition was also described for α-AI from kidney bean, using porcine pancreatic amylase [20]. It should be highlighted that in these experiments, starch was used as the substrate.

Our findings do not fully align with those previously reported by other authors, who described a non-competitive inhibition for α-AI of *P. vulgaris* var. Great Northern when using starch as the substrate [21]. A non-competitive mixed-type inhibition of a digestive amylase in the presence of a proteinaceous inhibitor from Moringa oleifera was also recently confirmed [33]. When comparing the data reported in the literature, there is no consensus on the type of amylase inhibition exhibited by protein inhibitors of plant origin. This discrepancy is not surprising considering the complex mechanism that results in the release of different peptides with amylase inhibitory activity.

Furthermore, the different type of reversible inhibition we observed may be due to the different substrate we used in our enzymatic assay. In fact, a mixed non-competitive inhibition suggests that the substrate interacts with the enzyme at more than one site. Therefore, it would not be surprising if different substrates could lead to different kinetic results.

## 3. Materials and Methods

### 3.1. Chemicals

2-Chloro-4-nitrophenyl-α-D-maltotrioside (93834-100MG), *p*-nitrophenyl-α-D-glucopyranoside (487506-1GM), α-amylase from porcine pancreas (Type VI-B A3176-2.5MU), PNGase F from *Elizabethkingia miricola* (P7367) and α-Glucosidase from *Saccharomyces cerevisiae* (Type I G5003-1KU) were purchased from Sigma–Aldrich (Milan, Italy). All other reagents used were of the highest grade available, purchased from Sigma–Aldrich (Milan, Italy) and used without further purification.

### 3.2. Plant Materials

The Sardinian *P. vulgaris* cultivar used to purify and characterize the inhibitor is called Nieddone, coming from Ploaghe, a Sardinian village. It is part of a wider germplasm collection of the Sardinian Agricultural Agency (AGRIS, Cagliari, Italy) collected with the accession number ACC.177. The α-AI partial mRNA sequence has been deposited in NCBI GenBank with the code OP530245.1.

### 3.3. Purification of Amylase Inhibitor

Legume seeds were homogenized to a fine powdery flour in a blender which was then immediately used for the extraction. Briefly, aliquots of 2 g were suspended in 10 mL 10 mM Bis-Tris buffer pH 6.5, 0.1 M NaCl buffer, then stirred for 1 h and centrifuged at 8000× *g* for 10 min at 4 °C. The extraction was also performed in the presence of 1 mM phenylmethylsulfonyl fluoride (PMSF) as a protease inhibitor.

The supernatant after extraction was collected and heated to denature heat-labile proteins for 10 min at difference temperatures and then centrifuged at 8000× *g* for 30 min at 4 °C. Alternatively, it was brought to 45–80% saturation with ammonium sulphate and centrifuged at 18,000× *g* for 30 min at 4 °C. Solvent precipitation was also used adding, dropwise, the raw extract to stirring iced solvent (ethanol or acetone) in a 1:50 volume ratio. The solution was then centrifuged at 8000× *g* for 20 min at 4 °C and the supernatant discarded. After 24 h (during which the residual solvent evaporated), the pellet was resuspended in 10 mL of starting buffer.

### 3.4. Ion Exchange Chromatography

The sample was applied to fast protein liquid chromatography (FPLC) in Akta Start (Cytiva, Milan, Italy) using anion exchange chromatography with HiTrap Q HP 5 mL column Start (Cytiva). Column was equilibrated with 5 column volumes of 10 mM Bis-Tris pH 6.5, 0.1 M NaCl buffer at 4 mL/min flow rate. The sample (8 mL) was injected, and unbound proteins were washed with column volumes buffer. Elution was then performed with a 20% step gradient in B buffer (10 mM Bis-Tris pH 6.5, 1 M NaCl), and 1 mL fractions were collected. After 4 column volumes, a linear gradient 100% B in 2 column volumes was then used to wash the column.

### 3.5. Size Exclusion Chromatography

A HiLoad 16/60 Superdex 200 pg (Cytiva) column was used (120 mL column volume). This was equilibrated with 4 column volumes of 10 mM Bis-Tris pH 6.5, 0.1 M NaCl, and 5 mM CaCl_2_ buffer at a 1 mL/min flow rate. The sample (4 mL) was injected, and 1 mL fractions were collected automatically in isocratic elution. The MW of the purified protein was determined by comparing with the MW standards (Gel Filtration Standards, cat. n. 1511901, Bio-Rad, Milan, Italy) under the same operational conditions. The purity of the sample was verified by total protein determination using the Bradford method [46] and by SDS-PAGE using 12% polyacrylamide gels using the Laemmli protocol [47].

### 3.6. α-Amylase and α-Glucosidase Inhibition Assays

The inhibition of α-amylase activity was determined using 2-chloro-p-nitrophenyl-α-D-maltotrioside (CNP-G3) as a substrate, as already described [4]. Briefly, 5 EU α-amylase from porcine pancreas (unless otherwise stated) were incubated in 250 mM sodium phosphate buffer at pH 6.5, 60 mM NaCl, 5 mM CaCl_2_ and 500 mM KSCN at 37 °C in a final volume of 150 μL. After 10′, 50 μL of a 9 mM CNP-G3 solution was added. The microplate spectrophotometer UV/VIS MultiskanGo (Thermo Fisher Scientific, Monza, Italy) was then used to quantify at 405 nm the substrate hydrolysis and consequent release of 2-chloro-*p*-nitrophenol [4]. Proper negative controls were performed in the absence of the enzyme, sample or substrate. As a positive control, a commercial food supplement extract (based on *P. vulgaris*) was added in each analysis.

One amylase E.U. was defined as the amount of enzyme capable of hydrolyze 1 μmol of CNP-G3 per minute at pH 6.5 and 37 °C (monitoring CNP formation, ε_405_ = 14,580 M^−1^ cm^−1^). The amylase inhibitory unit (IAU) was defined as the number of amylase units inhibited under the assay conditions.

The α-glucosidase inhibition assay used the same experimental conditions of the CNP-G3 protocol. The substrate used was *p*-nitrophenyl-α-D-glucopyranoside (pNPG) at 2.25 mM.

### 3.7. Hemagglutination Assay

Human blood, 2 mL, was centrifuged at 2000× *g* for 3.5 min, the supernatant was discarded and the cell pellet was washed three times with PBS buffer (137 mM NaCl, 2.7 mM KCl, 8 mM Na_2_HPO_4_, 1.5 mM KH_2_PO_4_, pH 7.4). The red blood cells (RBCs) pellet was suspended to 50% (*v*/*v*) with the PBS buffer, stabilized with 0.01% natrium azide and stored at 4 °C. The life span of RBCs was up to three weeks. One aliquot of RBCs, 10%, was prepared, and each raw extract was used at different serial dilutions (pure, 1/2, 1/4, 1/8, 1/16…as far as 1/512.). For each combination of RBCs and protein concentration, the RBC sample was pipetted onto a glass microscopy slide and mixed in a 10 μL:10 μL ratio with the protein solution. The mixture was incubated for 5 min and observed using an inverted fluorescence microscope BX81 (Olympus, Segrate, Italy). Images were captured with a charge-coupled device camera (Cohu, San Diego, CA, USA). In the control experiment, 10 μL of the PBS buffer was incubated with RBCs. The results are expressed as the minimum protein concentration able to Agglutinate sample (MAC, mg/mL).

### 3.8. α-Amylase from Tenebrio Molitor

Insect α-amylase was obtained from *Tenebrio molitor* larvae. This insect feeds on stored grains and food in general, and is usually considered a pest. Aliquots of 1 g of larvae were ground with 6 mL of 10 mM Bis-Tris buffer pH 6.5, 0.1 M NaCl, as already described [29]. This homogenate was then centrifuged at 8000× *g* for 30′. The supernatant was used as crude insect α-amylase preparation for activity assays as described above.

### 3.9. Inhibitor Characterization

The activity at different pH values was determined repeating the catalytic assay in the presence of different 0.5 M McIlvaine buffers ranging from pH 3 to 9 at 37 °C. Thermostability was assayed by incubating the enzyme at the indicated temperature for the indicated time. Then, the samples were rapidly brought to 4 °C and the remaining catalytic activity was assayed as previously described. Michaelis–Menten kinetic parameters were calculated using CNP-G3 ranging from 0.1 to 5 mM and using GraFit 7 (Erithacus Software, London, UK).

### 3.10. Deglycosylation and Preparation for MS Analysis

Deglycosylation was performed according to manufacture instructions. An aliquot of 50 μg protein was denatured with 0.2% SDS and 100 mM 2-mercaptoethanol at 100 °C for 10′. Then, 5 EU of PNGase F was added. After 3 h incubation at 37 °C, the reaction was stopped by heating at 100 °C for 5′.

The amylase-enriched sample (10 µL with a concentration of 1.5 µg/µL) was mixed 1:1 (*v*/*v*) with 60 mM Tris/HCl, pH 6.8, containing 2% SDS, 20% glycerol and 0.02% bromophenol blue with 2% 2-mercaptoethanol to perform SDS-PAGE under reducing conditions. Electrophoretic separation was performed in triplicate with the Mini-PROTEAN Tetra cell (Bio-Rad) at 180 V, and Precision Plus Protein Dual Color Standards (Bio-Rad) were used as molecular weight standards. The gel was stained with Bio-Safe TM Coomassie G250 stain (Bio-Rad), and then the slices corresponding to the protein of interest were excised. For destaining and peptide extraction procedures, the protocol of Gundry et al. was applied [48]. Tryptic peptides were dried, desalted with Pierce C18 zip tip (Thermo Fisher Scientific) following the manufacturer’s instructions, and finally, resuspended in 0.1% FA prior to the nano reverse-phase high-performance liquid chromatography (nano-RP-HPLC) coupled with high-resolution mass spectrometry (HR-MS/MS).

### 3.11. Nano-RP-HPLC–High-Resolution ESI-MS/MS Analysis

The characterization of the tryptic peptides extracted from the gel slices was obtained by nano-RP-HPLC-HR-MS/MS. The injected volume was 10 μL with a final concentration of 0.1 μg/μL. The analysis was performed with an Ultimate 3000 Nano System HPLC (Dionex-Thermo Fisher Scientific) coupled with an LTQ Orbitrap Elite (Thermo Fisher Scientific). The Easy Spray reverse-phase nano column (250 mm × 75 μm inner diameter, Thermo Fisher Scientific) was a C18 with 2 μm beads, and the elution of proteins and peptides was achieved with aqueous solvent A (0.1% FA) and aqueous solvent B (0.1% FA, 80% Acetonitrile *v*/*v*) in 100 min at a flow rate of 0.3 μL/min with the following gradient: 0–3 min at 4%B, 3–80 min 4–50%B, 80–90 min 80–90%B, 90–92 min 90%B and 92–100 min 90%B. The mass spectrometer operated at 1.5 kV in the data-dependent acquisition mode, with the capillary temperature set at 275 °C and S-Lens RF level 68.0%. Full MS experiments were performed in positive ion mode from 350 to 1600 *m*/*z* with resolution 120,000 (at 400 *m*/*z*). The 10 most intense ions were subjected to CID fragmentation with settings of 35% of normalized collision energy for 10 ms, an isolation width of 2 *m*/*z* and activation q of 0.25. Spectra were analyzed with the help of Proteome Discoverer (PD) software (version 2.2, Thermo Fisher) with the SEQUEST HT cluster search engine (University of Washington, licensed to Thermo Electron Corporation, San Jose, CA, USA) against the target and reviewed list of proteins from Phaseolus vulgaris available from the UniprotKB database (325 entries, April 2023). The database search parameters were the dynamic acetylation at N-terminal and Lysin residue, and the carbamido methylation of cysteine as a stable modification. Peptide mass tolerance was set to 10 ppm and the fragment ion mass tolerance was set to 0.6 Da. Proteins were filtered for high confidence, with at least 2 unique peptides and a coverage percentage equal or greater than 30%; FDR settings were 0.01 (strict) and 0.05 (relaxed).

To establish whether the sequence of the unique tryptic peptides identified after PD software analysis were shared with any organism different from *Phaseolus vulgaris*, we performed an analysis with the bioinformatic tool BLAST (freely available at https://www.uniprot.org/blast, accessed on 16 July 2024) with the following settings: Sequence type “protein”, Program “blastp”, E-threshold “1”, Matrix “Auto-BLOSUM62” and “no gap” within the query sequence. To let the tool perform the search against all the protein sequences from all known organisms available in the repository UniprotKB, no taxonomy restriction was applied but only reviewed proteins were accepted.

### 3.12. Statistical Analysis

GraFit 7 (Erithacus Software, London, UK), R 2.5.1 (R Foundation for Statistical Computing, Vienna, Austria) and GraphPad INSTAT 8.2.1 (GraphPad Software, San Diego, CA, USA) were used for data analysis. One-way analysis of variance (ANOVA) and the Bonferroni multiple comparison test were used to assess the statistical significance of the differences. All the analyses were performed at least in triplicate (unless otherwise stated), and the data are reported as mean ± standard error of the mean (SEM).

## 4. Conclusions

Using low-resolution techniques and chromatographic steps, the α-amylase inhibitor has been successfully purified from the common bean seeds (*Phaseolus vulgaris*) of the Sardinian cultivar Nieddone. The tetrameric structure of the inhibitor, with a molecular weight of approximately 42 kDa, confirms that this protein is a lectin-like α-AI inhibitor, with the typical α_2_β_2_ association that has been previously reported in the literature. This successful purification and confirmation of the structure provide a solid foundation for further studies and potential applications of this inhibitor.

Indeed, the α-amylase inhibitor has demonstrated its ability to maintain its activity over time, even when exposed to high temperatures up to 100 °C, and across various pH values. This resilience to high temperatures and stability across a range of pH levels make it an effective component in food supplements designed to withstand diverse gut conditions. Moreover, its compatibility with cooked food production processes opens opportunities for enriching various food items with α-AI, offering consumers an innovative way to meet their dietary requirements and preferences.

The α-AI has proven to be effective not only against human α-amylase but also against the α-amylase enzyme found in pigs and insects. This broad range of activity opens new possibilities for pest control and combating insect infestation.

Certainly, before proceeding with commercial applications of a cultivar such as Nieddone, it is crucial to thoroughly investigate the feasibility of extensive cultivation. This is, especially important considering its current limited cultivation in small plots of land within restricted spatial regions. Several factors need to be considered, including agronomic aspects, genetic integrity, resistance to local pathogens and other relevant factors to ensure successful cultivation and subsequent commercialization.

By delving deeper into these aspects, we not only pave the way for potential commercial applications but also contribute to the conservation and valorization of Sardinian biodiversity. Understanding the unique characteristics of both the proteins derived from these cultivars and the cultivars themselves can lead to sustainable agricultural practices that not only preserve but also utilize the rich biodiversity of Sardinia. This holistic approach ensures that any commercial endeavors are rooted in a thorough understanding of the ecological and agricultural context, promoting both economic development and environmental conservation.

## Figures and Tables

**Table 1:** Purification table of *P. vulgaris* Nieddone cv α-AI.

	Amylase Inhibiting Activity (IAU)	Total Protein (mg)	Recovery	Specific Activity (IAU/mg)	Purification Fold
Raw extract	242	60.22	100%	4	1.0
Solvent precipitation	147	15.68	61%	9	2.3
IEC	59	3.24	24%	18	4.5
SEC	80	0.24	33%	328	81.8

**Table 2:** Determination of kinetics parameters for α-AI purified from Nieddone cv.CNP-G3 used as substrate.

	α-AI = 0	α-AI = 0.25 μM	α-AI = 0.50 μM
Vmax (µM/min)	30.9	28.8	21.8
K_M_ (mM)	0.85	1.01	1.10

## Data Availability

Data is contained within the article.

## Supplementary Materials

The following supporting information can be downloaded at: https://www.mdpi.com/article/10.3390/plants13152074/s1, Figure S1: α-AI activity recorded after different extraction conditions; Figure S2: Comparison of % recovery of α-AI extraction after ammonium sulfate saturation and organic solvent precipitation; Figure S3: Annotated MS/MS spectra from Proteome Discoverer software for each tryptic peptide of α-AI from *P. vulgaris* Nieddone cultivar. Panels A to G refer to each tryptic peptide structural characterization by the attribution of b, a and y ions based on matching between the theoretical and experimental MS/MS fragmentation; Figure S4: BLAST analysis of the tryptic peptides identified. Fast family and domain prediction based on query sequence (the 7 tryptic peptides characterized by MS/MS analysis) (A) and alignment between the query sequence and the sequences of the two candidate proteins where the correspondence is highlighted in light blue (B). Figure S5: Calibration of SEC with MW standards to determine α-AI MW; Figure S6: Trend of inhibition of α amylase enzyme by α-AI with different preincubation times; Figure S7: Effect of pH on the activity of α-amylase enzyme and on the activity of the purified inhibitor from Nieddone cv.
