# Supplementary material for: Purification and Characterization of Proteinaceous Thermostable α-Amylase Inhibitor from Sardinian Common Bean Nieddone Cultivar (Phaseolus vulgaris L.)
Source: Plants (Basel). 2024 Jul 26;13(15):2074. doi: 10.3390/plants13152074 (PMC11314397; doi:10.3390/plants13152074)
Supplement: Supplementary file 1 [file plants-13-02074-s001.zip › plants-3044945-supplementary.pdf]

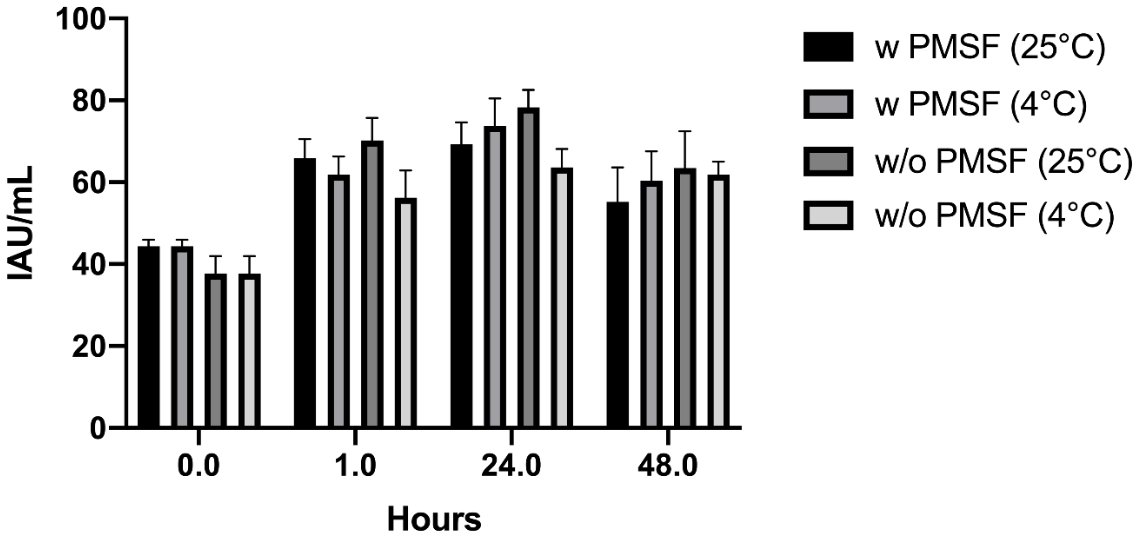

Figure S1. α-AI activity recorded after different extraction conditions.

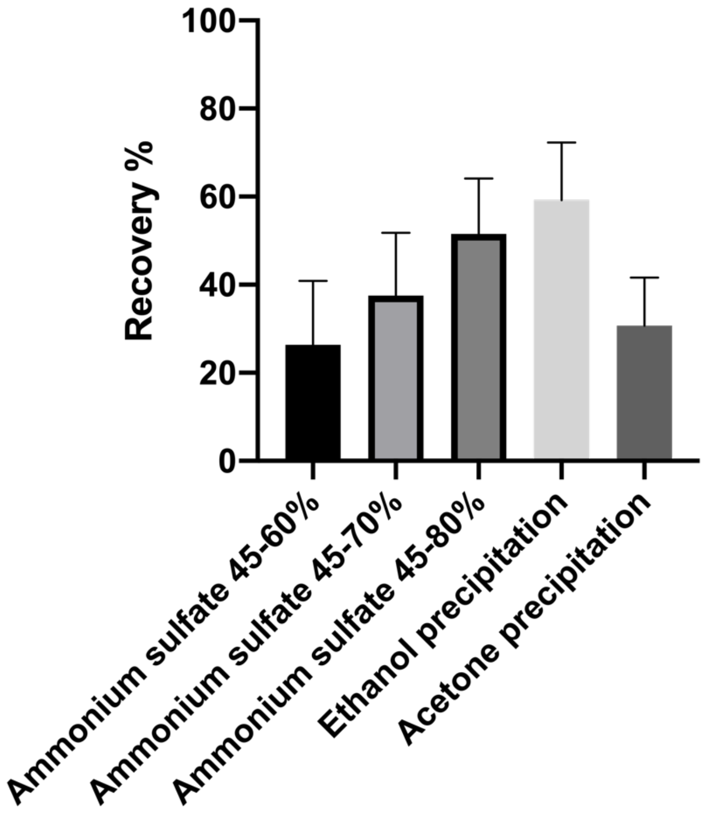

Figure S2. *Comparison of recovery % α-AI extraction after ammonium sulfate saturation and organic solvent precipitation.*

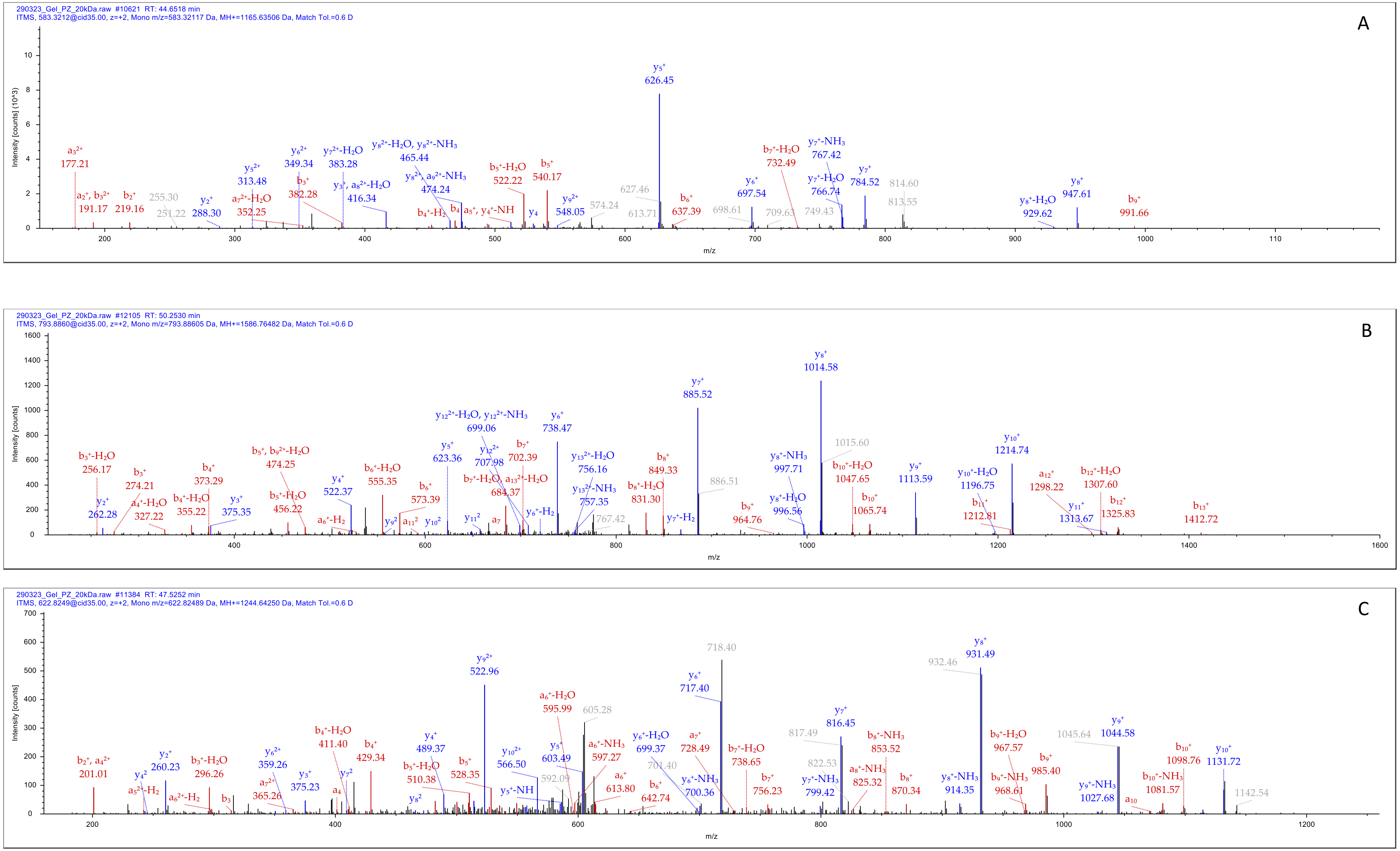
290323_Gel_PZ_20kDa.raw #10621 RT: 44.6518 min
ITMS, 583.3212@cid35.00, z=+2, Mono m/z=583.32117 Da, MH+=1165.63506 Da, Match Tol.=0.6 D
A
Intensity [counts] (10^3)
m/z
y5+ 626.45
a3 2+ 177.21
a2+, b3 2+ 191.17
b2+ 219.16
255.30
251.22
y2+ 288.30
y5 2+ 313.48
a7 2+-H2O 352.25
y6 2+ 349.34
b3+ 382.28
y7 2+-H2O 383.28
y3+, a8 2+-H2O 416.34
y8 2+-H2O, y8 2+-NH3 465.44
y8 2+, a9 2+-NH3 474.24
b4+-H2
b4
a5+, y4+-NH
b5+-H2O 522.22
b5+ 540.17
y9 2+ 548.05
574.24
627.46
613.71
b6+ 637.39
698.61
y6+ 697.54
709.63
b7+-H2O 732.49
749.43
y7+-NH3 767.42
y7+-H2O 766.74
y7+ 784.52
814.60
813.55
y8+-H2O 929.62
y8+ 947.61
b9+ 991.66
290323_Gel_PZ_20kDa.raw #12105 RT: 50.2530 min
ITMS, 793.8860@cid35.00, z=+2, Mono m/z=793.88605 Da, MH+=1586.76482 Da, Match Tol.=0.6 D
B
Intensity [counts]
m/z
b3+-H2O 256.17
y2+ 262.28
b3+ 274.21
a4+-H2O 327.22
b4+-H2O 355.22
b4+ 373.29
y3+ 375.35
b5+, b9 2+-H2O 474.25
b5+-H2O 456.22
y4+ 522.37
a6+-H2
b6+-H2O 555.35
b6+ 573.39
y5+ 623.36
y12 2+-H2O, y12 2+-NH3 699.06
y12 2+ 707.98
b7+ 702.39
b7+-H2O, a13 2+-H2O 684.37
y6+ 738.47
y6+-H2
y13 2+-H2O 756.16
y13 2+-NH3 757.35
767.42
b8+ 849.33
b8+-H2O 831.30
y7+-H2
y7+ 885.52
886.51
b9+ 964.76
y8+-NH3 997.71
y8+-H2O 996.56
y8+ 1014.58
1015.60
b10+-H2O 1047.65
b10+ 1065.74
y9+ 1113.59
y10+-H2O 1196.75
b11+ 1212.81
y10+ 1214.74
a12+ 1298.22
b12+-H2O 1307.60
y11+ 1313.67
b12+ 1325.83
b13+ 1412.72
290323_Gel_PZ_20kDa.raw #11384 RT: 47.5252 min
ITMS, 622.8249@cid35.00, z=+2, Mono m/z=622.82489 Da, MH+=1244.64250 Da, Match Tol.=0.6 D
C
Intensity [counts]
m/z
b2+, a4 2+ 201.01
y2+ 260.23
a5 2+-H2
a6 2+-H2
b3+-H2O 296.26
b3
y6 2+ 359.26
a7 2+ 365.26
y3+ 375.23
b4+-H2O 411.40
b4+ 429.34
y4+ 489.37
b5+-H2O 510.38
b5+ 528.35
y9 2+ 522.96
y10 2+ 566.50
y5+-NH
y5+ 603.49
592.09
a6+-H2O 595.99
605.28
a6+-NH3 597.27
a6+ 613.80
b6+ 642.74
y6+-H2O 699.37
701.40
y6+-NH3 700.36
y6+ 717.40
718.40
a7+ 728.49
b7+-H2O 738.65
b7+ 756.23
817.49
y7+ 816.45
822.53
y7+-NH3 799.42
b8+-NH3 853.52
a8+-NH3 825.32
b8+ 870.34
y8+-NH3 914.35
932.46
y8+ 931.49
b9+-H2O 967.57
b9+-NH3 968.61
b9+ 985.40
1045.64
y9+ 1044.58
y9+-NH3 1027.68
a10
b10+-NH3 1081.57
b10+ 1098.76
y10+ 1131.72
1142.54

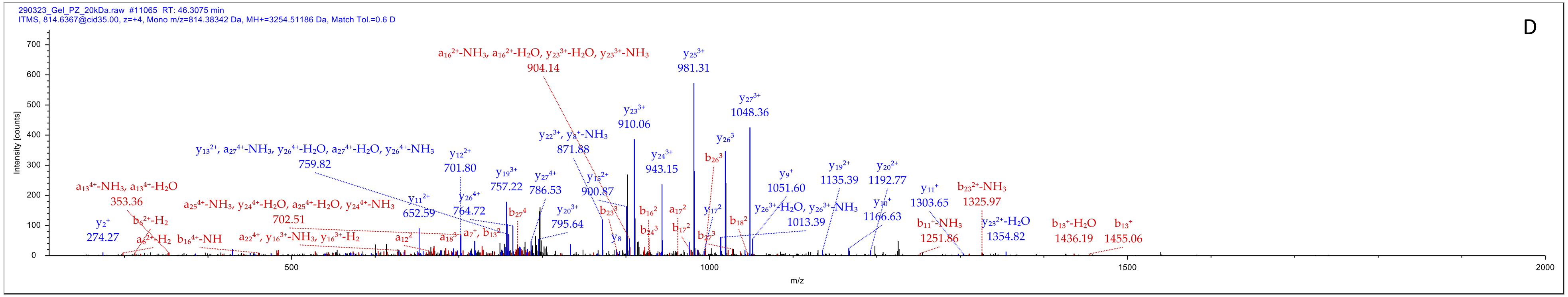
290323_Gel_PZ_20kDa.raw #11065 RT: 46.3075 min
ITMS, 814.6367@cid35.00, z=+4, Mono m/z=814.38342 Da, MH+=3254.51186 Da, Match Tol.=0.6 D
D
Intensity [counts]
m/z

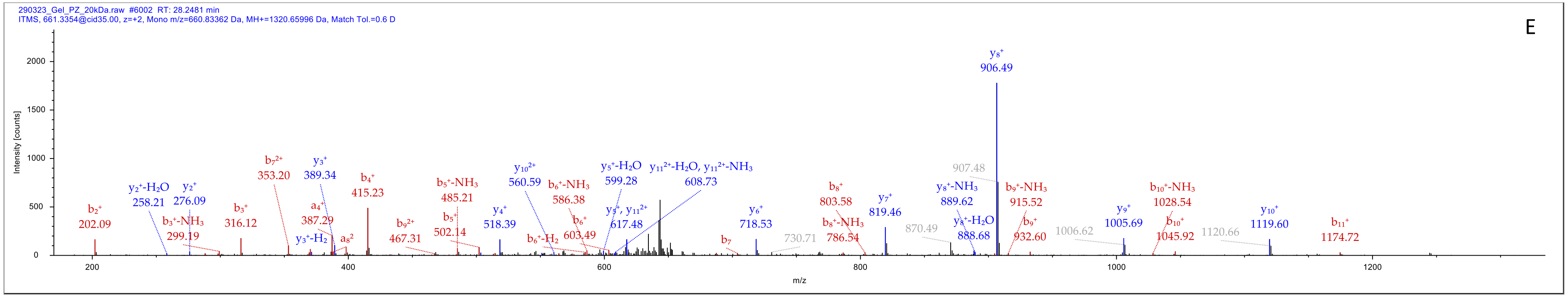
290323_Gel_PZ_20kDa.raw #6002 RT: 28.2481 min
ITMS, 661.3354@cid35.00, z=+2, Mono m/z=660.83362 Da, MH+=1320.65996 Da, Match Tol.=0.6 D
E
Intensity [counts]
m/z

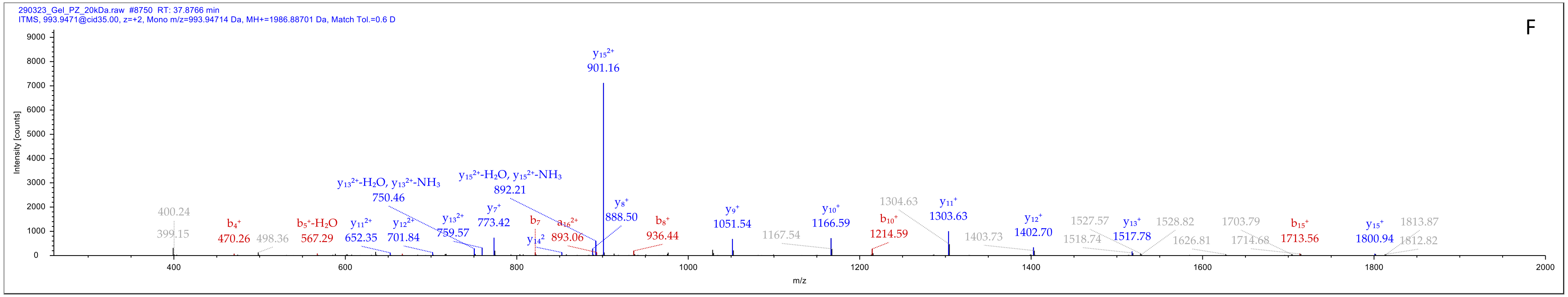
290323_Gel_PZ_20kDa.raw #8750 RT: 37.8766 min
ITMS, 993.9471@cid35.00, z=+2, Mono m/z=993.94714 Da, MH+=1986.88701 Da, Match Tol.=0.6 D
F
Intensity [counts]
m/z

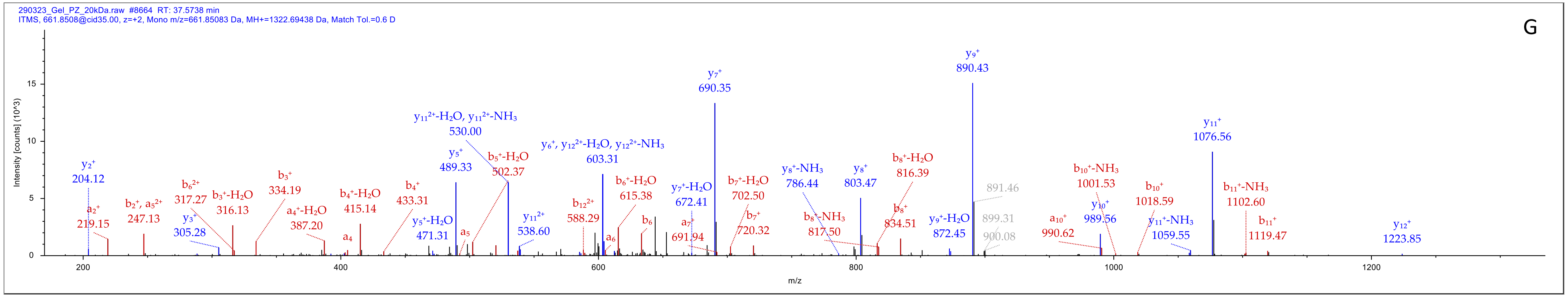

Figure S3. Annotated MS/MS spectra from Proteome Discoverer software for each tryptic peptide of α-AI from *P. vulgaris* Nieddone cultivar. Panels A to G refer to each tryptic peptide structural characterization by the attribution of b, a, and y ions based on the matching among the theoretical and experimental MS/MS fragmentation.

A

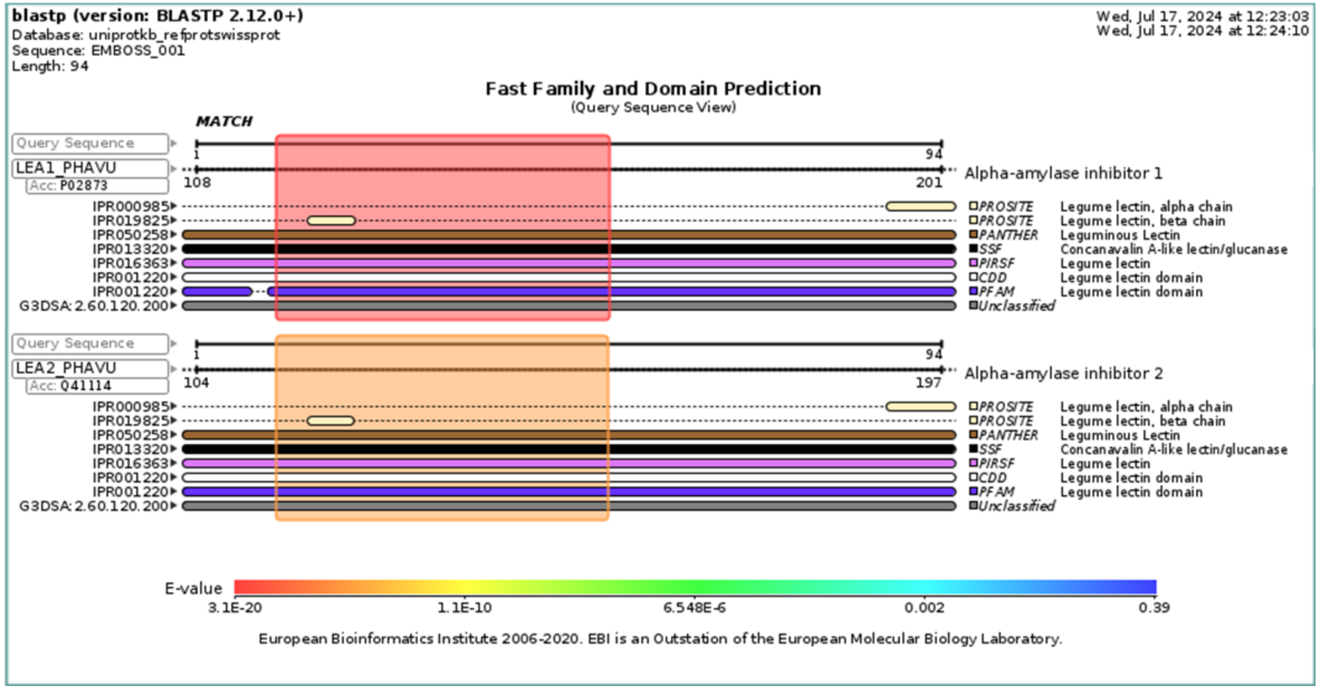

blastp (version: BLASTP 2.12.0+)
Database: uniprotkb_refprotswissprot
Sequence: EMBOSS_001
Length: 94
Wed, Jul 17, 2024 at 12:23:03
Wed, Jul 17, 2024 at 12:24:10
Fast Family and Domain Prediction
(Query Sequence View)
MATCH
Query Sequence
LEA1_PHAVU
Acc: P02873
Alpha-amylase inhibitor 1
IPR000985 PROSITE Legume lectin, alpha chain
IPR019825 PROSITE Legume lectin, beta chain
IPR050258 PANTHER Leguminous Lectin
IPR013320 SSF Concanavalin A-like lectin/glucanase
IPR016363 PIRSF Legume lectin
IPR001220 CDD Legume lectin domain
IPR001220 PFAM Legume lectin domain
G3DSA:2.60.120.200 Unclassified
LEA2_PHAVU
Acc: Q41114
Alpha-amylase inhibitor 2
E-value
3.1E-20
1.1E-10
6.548E-6
0.002
0.39
European Bioinformatics Institute 2006-2020. EBI is an Outstation of the European Molecular Biology Laboratory.

B

CLUSTAL O(1.2.4) multiple sequence alignment

```
Query
sp|Q41114|LEA2_PHAVU      ----MASSNLLTLALFLVLLTHANSASDTSFNFYSFNETNLILQGDATVSSKGYLQL--HTV        56
sp|P02873|LEA1_PHAVU      MIMASSKLLSLALFLALLSHANSATETSFIIDAFNKTNLILQGDATVSSNGNLQLSYNSY        60


Query                          AFYSAPIQIR                                     GDT
sp|Q41114|LEA2_PHAVU      DSMCSAFYSAPIQIRDSTTGNVASFDTNFTMNITTQRE ANSV IGLDFALVPVQPKSK GHT       116
sp|P02873|LEA1_PHAVU      DSMSRAFYSAPIQIRDSTTGNVASFDTNFTMNIRTHRQANSAVGLDFVLVPVQPESKGDT       120

Query                     VTVE FDTFL SRISIDVNNNDIKSVPWDVHDYDGQNAEVR
sp|Q41114|LEA2_PHAVU      VTVQFDTFRSRISIDVNNNDIKSVPWDEQDYDGQNAKVRIT---------YNSSTKVLAV        167
sp|P02873|LEA1_PHAVU      VTVEFDTF LSRISIDVNNNDIKSVPWDVHDYDGQNAEVRIT---------YNSSTKVFSV       171

Query                     SLSNPSTGKSNNVS T T VE LEK
sp|Q41114|LEA2_PHAVU      SLSNPSTGKSNE VSARMEVEKELDDWVRVGFSAISGVHEYSFETRDVLSWSFSSKFSQH-       226
sp|P02873|LEA1_PHAVU      SLSNPSTGKSNNVS T TVE LEKEVYDWVSVGFSATSGAYQWSYETHDVLSWSFSSKFINLK     231

Query
sp|Q41114|LEA2_PHAVU      --TTSERSNILLNNIL          240
sp|P02873|LEA1_PHAVU      DQKSERSNIVLNKIL           246
```

Figure S4. BLAST analysis of the tryptic peptides identified. Fast family and domain prediction based on query sequence (the 7 tryptic peptides characterized by MS/MS analysis) (A) and alignment between the query sequence and the sequences of the two candidate proteins where the correspondence is highlighted in light blue (B).

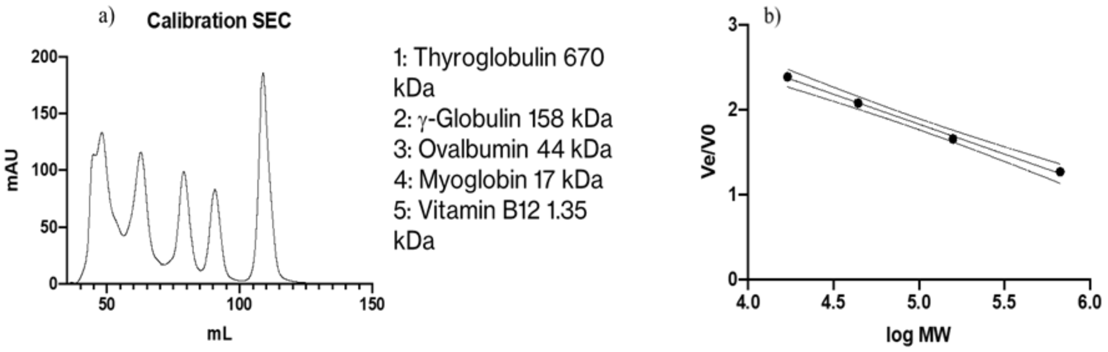

Figure S5. *Calibration of SEC with MW standards to determine α -AI MW.*

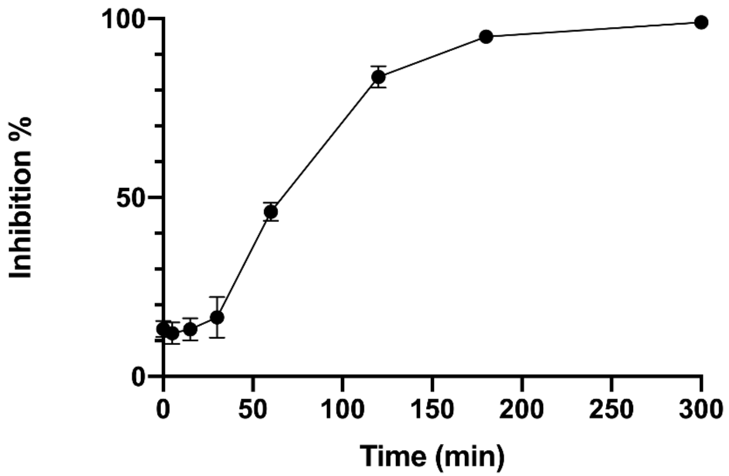

Figure S6. Trend of inihibition of α amylase enzyme by α-AI with different preincubation times.

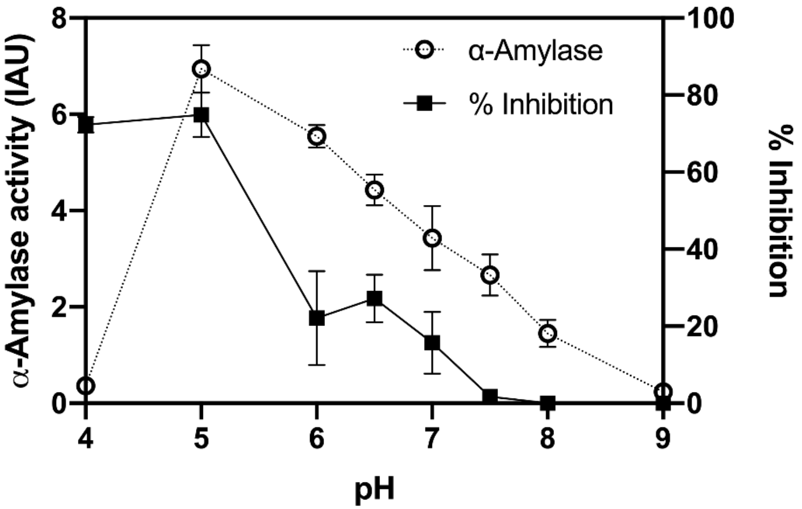

Figure S7. Effect of pH on the activity of α-amylase enzyme and on the activity of the purified inhibitor form Nieddone cv.
